# EP3 (prostaglandin E2 receptor 3) expression is a prognostic factor for progression-free and overall survival in sporadic breast cancer

**DOI:** 10.1186/s12885-018-4286-9

**Published:** 2018-04-16

**Authors:** Anna Semmlinger, Viktoria von Schoenfeldt, Verena Wolf, Alexandra Meuter, Theresa Maria Kolben, Thomas Kolben, Christine Zeder-Goess, Florian Weis, Julia Gallwas, Rachel Wuerstlein, Kerstin Hermelink, Elisa Schmoeckel, Nadia Harbeck, Doris Mayr, Sven Mahner, Udo Jeschke, Nina Ditsch

**Affiliations:** 1 Department of Obstetrics and Gynecology & Breast Center, University Hospital, LMU Munich, Marchioninistrasse 15, 81377 Munich, Germany; 2 Department of Obstetrics and Gynecology & Breast Center, University Hospital, LMU Munich, Maistrasse 11, 80337 Munich, Germany; 3 Department of Pathology, LMU Munich, Thalkirchnerstrasse 36, 80337 Munich, Germany; 4 Department of Anaesthesiology, Klinikum Fuerstenfeldbruck, Dachauer Strasse 33, 82256 Fuerstenfeldbruck, Germany

**Keywords:** Breast cancer, EP3, Prostaglandin E2, EP-receptor, COX-2, PGE2, Prognostic factor

## Abstract

**Background:**

In various cancers, overexpression of cyclooxygenase (COX)-2 and elevated prostaglandin (PG) E2 synthesis have been associated with tumor development and progression. The potential of COX-2 inhibitors in cancer prevention and treatment has been shown repeatedly; however, their clinical use is limited due to toxicity. PGE2 signals via EP receptors 1–4, whose functions are analyzed in current research in search for targeted anti-PG therapies. EP2 and EP4 rather promote tumorigenesis, while the role of EP3, especially in breast cancer, is not yet clear and both pro- and anti-tumorigenic effects have been described. Our study evaluates EP3 receptor expression in sporadic breast cancer and its association with clinicopathological parameters, progression-free and overall survival.

**Methods:**

Two hundred eighty-nine sporadic breast cancer samples without primary distant metastasis were immunohistochemically analyzed for EP3 receptor expression. Tissue was stained with primary anti-EP3-antibodies. Immunoreactivity was quantified by the immunoreactivity-score (IRS); samples with an IRS ≥ 2 scored as EP3 positive. Chi-squared and Mann-Whitney-U test were used for comparison of data; Kaplan-Meier estimates and Cox-regression were used for survival analyses.

**Results:**

EP3 receptor was expressed in 205 of 289 samples analyzed (70.9%). EP3 receptor expression was not associated with clinicopathological parameters (e. g. tumor size, hormone receptors, lymph node status). Kaplan-Meier estimates showed a significant association of EP3 positivity with improved progression-free survival (*p* = 0.002) and improved overall survival (*p* = 0.001) after up to 10 years. Cox regression analysis confirmed EP3 positivity as a significant prognostic factor even when other known prognosticators were accounted for.

**Conclusions:**

In sporadic breast cancer, EP3 receptor expression is not significantly associated with clinicopathological parameters but is a significant prognostic factor for improved progression-free and overall survival. However, the functional aspects of EP3 receptor in breast cancer and the way how EP3 may oppose the pro-tumorigenic effects of PGE2 elevation and COX-2 overexpression are not fully understood so far. Further studies aiming at identification of the factors regulated by EP3 are necessary to evaluate the possibility of targeting EP3 in future anti-tumor therapy in breast cancer.

**Electronic supplementary material:**

The online version of this article (10.1186/s12885-018-4286-9) contains supplementary material, which is available to authorized users.

## Background

Breast cancer is the most common female cancer (30.8% of all newly diagnosed cancers in women in Germany, 2012) and also the most common cause of cancer-related death in women [1]. The development of treatment from conventional to targeted anti-tumor therapies, e. g. human epidermal growth factor receptor 2 (HER2) blockade, has contributed much to improve breast cancer therapy [2]. However, as these therapies are not suitable for all patients, the search for new, individualized and specific alternatives of treatment is ongoing.

Eicosanoides, which include leukotriens and prostaglandins (PG), are tissue hormones that can act in an autocrine or paracrine manner. They contribute to uncountable physiological and pathological processes: The balance of thromboxane A2 and prostacyclin e. g. is essential for hemostasis, whereas PGE_2_ is the most abundant prostaglandin in humans and is known as a key mediator in inflammation. Cyclooxygenase (COX) enzymes are the primary enzymes in the synthesis of eicosanoides and exist in two isoforms: COX-1 is considered to be ubiquitously expressed, whereas COX-2 is normally absent from most human tissues. COX-2 upregulation can be elicited by various cytokines and growth factors and is involved in the regulation of the inflammatory response. Both COX-enzymes catalyze the conversion of arachidonic acid to PGG_2_ and subsequently to PGH_2_, which itself is the precursor molecule for the synthesis of the different eicosanoids, including PGE_2_, prostacyclin or thromboxane A_2_ [3, 4].

Besides its known essential function in physiological processes and inflammation, PGE_2_ plays an important role in tumorigenesis: In various cancer samples, elevated PGE_2_ levels have been found. Similarly, constitutive COX-2 overexpression - which is thought to be responsible for the increase in PGE_2_ levels - has been detected in various tumors [5]. Both increased PGE_2_ and COX-2 overexpression have been associated with tumorigenesis and progression [3, 6]. Therefore, the role of prostaglandins and their mediators in tumorigenesis and their potential as possible therapeutic targets have come into focus of recent research [4, 7].

In breast cancer, COX-2 overexpression is found in about 40% of all cases, it is inversely associated with survival rates and is positively associated with various characteristics of aggressive disease [3, 6]. Studies in murine and human breast cancer models have shown the influence of COX-2 overexpression on breast tumor development, e. g. via the mechanisms of inducing angiogenesis or improving cell mobility and invasiveness [8, 9]. Experimental blockade of COX-2 in a mouse model has reduced tumor progression [10]. Similarly, COX-2 antagonist (COXib) treatment in humans has shown an impressive preventive effect with a significant reduction in the risk of breast cancer in a case control study [11]. These strikingly beneficial effects of inhibiting COX-2 show the potential of the COX-2-PGE_2_-axis in breast cancer prevention and therapy. Unfortunately, the use of COXibs in clinical practice is limited due to their strong cardiovascular toxicity (thrombosis, atherogenesis, hypertension). The side effects are thought to be caused by the selective depression of prostacyclin synthesis (via COX-2 inhibition), whereas thromboxane A_2_ synthesis via COX-1 is not influenced and in consequence, the balance between pro- and antithrombotic agents is disturbed [3]. Therefore, elucidating the role of other components involved in the exertion of PGE_2_-effects in search for targeted breast cancer therapies with lesser side effects has come into focus of research.

PGE_2_ mediates its effects via four G-protein coupled receptors, the EP receptors 1–4 with different intracellular signaling pathways [4, 12]. EP2 and EP4 receptor are coupled to Gs-protein/protein kinase A/adenylate cyclase and induce intracellular elevation of cyclic adenosine monophosphate (cAMP). EP1 receptor is associated to Gq-protein and mediates elevation of intracellular calcium and phospholipase C activation [7, 12]. As a remarkable feature, EP3 (Prostaglandin E2 receptor 3) exists in multiple isoforms (generated by alternative mRNA splicing); eight different isoforms are identified in humans and three isoforms in mice signaling via different G-proteins. Most human EP3 isoforms inhibit cAMP generation via Gi-protein (on the contrary to EP2 and EP4 which increase cAMP levels), some isoforms can also increase intracellular calcium like EP1, some might also signal via Gs proteins [4].

EP1–4 receptor expression has been shown in a variety of cancers, including breast cancer [13–15]. Exploiting the role of EP receptors in breast cancer tumorigenesis, it has been found that in COX-2-induced murine mammary tumors, EP1, 2 and 4 are strongly induced compared to normal mammary gland tissue, whereas EP3 receptor expression is rather downregulated, but still detectable [8]. However, concerning the prognostic relevance of EP receptor expression and the effects of receptor blockade or stimulation on breast cancer development and the course of clinical disease, studies partly show different effects (reviewed in [4, 7] and [16]). Most data is available concerning EP2 and EP4, whose elevated expression in mammary tumor cells is mostly associated with enhanced metastasis, tumor cell proliferation and tumor invasiveness [13, 14, 17]. Limited data is available concerning EP1 in breast cancer: It has been associated with tumor development [7, 18], but another study associated EP1 expression with suppression of metastasis while it had no effect on the primary tumor and EP1 positive patients had improved survival [19]. Therefore, EP1 might have a pro-tumorigenic effect on the primary tumor but might also have anti-metastatic effects in breast cancer [19]. Of the EP receptors, EP3 is the least well-understood in breast cancer with both tumor-promoting and suppressive effects having been published [4].

As EP3 seems to have a different role than the other EP receptors – EP3 is downregulated in tumor cells and shows rather inhibitory signaling mechanisms – it is suggesting that EP3 might have a protective role in mammary tumor development and its expression on cancer cells might be associated with a more favorable course of the disease. This makes EP3 an interesting target to analyze and might also open the possibility to target EP3 in future specific cancer therapy.

To our knowledge, no sufficient data exists concerning prognostic relevance of EP3 in sporadic breast cancer. Therefore, we performed this study to evaluate the expression of EP3 receptor in sporadic breast cancer and its association with clinicopathological parameters (tumor size, lymph node status, focality, grading, hormone receptors, HER2-amplification, age), progression and survival.

## Methods

### Patients

In this study, 320 consecutive patients who underwent surgery for breast cancer from 2000 to 2002 at the Department of Gynaecology and Obstetrics, Ludwig-Maximilians-University of Munich, Germany and of which tumor tissue was still available were included. Only cases with a diagnosis of sporadic breast cancer without family history for breast cancer were included. Patients with primary distant metastasis were excluded from further analysis. The histological type and tumor grading (according to the Elston-Ellis system) was assigned by the institute of pathology, Ludwig-Maximilians-University of Munich; tumors were classified according to the TNM staging system. Patient data regarding patient age, hormone receptor status (estrogen receptor [ER], progesterone receptor [PR]), HER2-amplification, metastasis, local recurrence, progression and survival were retrieved from the Munich Cancer Registry.

Progression-free and overall survival was statistically analyzed after an observation period of up to 10 years.

### Immunohistochemistry

Directly after resection, breast cancer tissue specimens were fixated in formalin solution and were then embedded in paraffin. Immunohistochemistry was performed as described previously [20]. Primary anti-EP3-antibodies (polyclonal rabbit IgG, Abcam, Cambridge, UK) were used for tissue slide staining and detected via polymer-method (ZytoChem Plus HRP Polymer System mouse/rabbit, Zytomed Systems Berlin, Germany) and the chromogen diaminobenzidine (Dako, Hamburg, Germany). Placenta tissue served as positive control, normal serum served as a negative control. Samples were then analyzed by the semi-quantitative immunoreactivity score (IRS) using a Leitz (Wetzlar, Germany) microscope. The IRS multiplies the intensity of the staining (0 = no, 1 = weak, 2 = moderate, 3 = strong staining) with the percentage of positive cells (0 = no staining, 1 = < 10% positive cells, 2 = 11–50% positive cells, 3 ≥ 50% positive cells). According to their IRS, samples were then classified as EP3 positive or EP3 negative: Samples with an IRS of 0 or 1 were counted as EP3 negative, samples with an IRS of 2 or higher were counted as EP3 positive. Both groups were then compared for clinicopathological parameters, progression-free and overall survival.

### Statistics

IBM SPSS software version 24 was used to analyze data. MicrosoftExcel 2010 was used for illustrations. *P*-values ≤0.05 were considered statistically significant. Chi-squared test was used to determine independence between nominal data, Mann-Whitney-U test was used to analyze continuous variables. Kaplan-Meier estimates were used to estimate progression-free and overall survival in EP3 positive and EP3 negative patient groups; survival distributions were compared by the log-rank-test. Cox regression analysis was performed to account for effects of further variables. In the multivariate analysis, we included EP3 expression and patient age and other clinicopathological factors which had shown *p*-values ≤0.10 in bivariate survival analyses. These were namely tumor size, lymph node status, ER, PR and HER2 for overall survival and tumor size, lymph node status and ER for progression free survival. Assumptions of the Cox regression, namely non-informative censoring and proportional hazards, were met in all analyses.

## Results

### Patient characteristics

From the 320 consecutive patients included in this study, successful EP3 staining and sufficient follow-up data could be obtained from 289 patients. Median patient age was 58 years (range 69). 49.8% of all patients had carcinoma of no special type (NST). 70.9% of all patients had a G1 or G2 tumor, however, tumor grading could only be obtained in 151/289 patients and the influence of this parameter therefore has to be regarded with limited reliability. Because of limited data we did also decide not to include grading in Cox regression analyses. The majority of patients had a breast tumor smaller than 2 cm in size (pT1: 67.1%, pT2: 28.4%, pT3: 1%, pT4 3.4%) and most patients did not have primary lymph node metastasis (pN0: 57.5% of all patients). However, 42.5% of all patients had lymph node metastasis, 47.8% of all had more than one focus. Most patients were positive for ER (81%) and PR (58.8%). HER2 was not amplificated in 87.5% of all patients. Patient characteristics are displayed in Table 1. Total patient numbers in each subgroup differ because information regarding each subgroup could not be obtained from each patient.

**Table 1:** Patient characteristics

		n	(%)			n	(%)
histology				tumor grade			
	NST	144	(49.8)		G1-G2	107	(70.9)
	non NST	145	(50.2)		G3	44	(29.1)
tumor foci				PR			
	unifocal	151	(52.2)		negative	119	(41.2)
	≥ 2 foci	138	(47.8)		positive	170	(58.8)
pT				HER2			
	pT1	193	(66.8)		negative	246	(87.5)
	pT2-pT4	96	(33.2)		positive	35	(12.5)
pN				age (years)			
	pN0	165	(57.5)		median	58.0	
	pN1-pN3	122	(42.5)		range	69	
ER							
	negative	55	(19.0)				
	positive	234	(81.0)				

*NST* no special type, *ER* estrogen receptor, *PR* progesteron receptor

Follow-up data could be obtained from 147/289 patients for the complete observation period of 10 years with a median follow-up for the whole cohort of 10 years (range 9.92 years).

### EP3 staining is independent of clinicopathological parameters

**Fig. 1:**
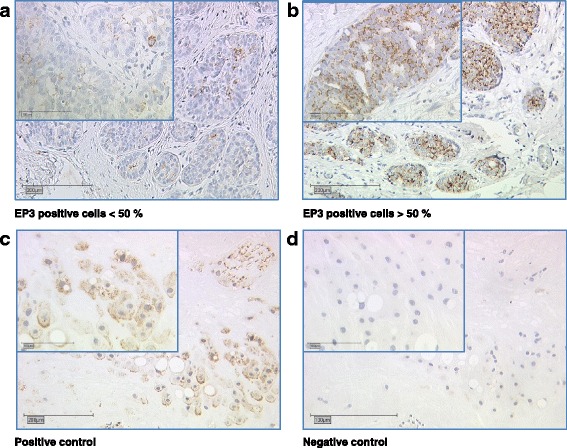
EP3 expression in breast cancer. Exemplary immuno-histochemical EP3 staining of breast cancer tissue samples is displayed. **a** Percentage of EP3 positive cells < 50%. **b** Percentage of EP3 positive cells > 50%. **c** Positive control (placenta). **d** Negative control (normal serum)

EP3 staining was positive in 70.9% (205/289) breast cancer tissue sections. Examples of tissue sections stained for EP3 are displayed in Fig. 1, displaying one sample with low (Fig. 1a) and one sample with high (Fig. 1b) percentage of EP3 positive cells. Furthermore, positive (Fig. 1c) and negative (Fig. 1d) staining controls are shown.

No associations of EP3 expression with clinicopathological parameters (which are listed in Table 1) were found. EP3 expression could be shown in all histological subtypes of breast cancer. Patient age at diagnosis did not differ between EP3 positive and EP3 negative patient groups (median age 59 years in EP3 negative and 57.7 years in EP3 positive patients, *p* = 0.286). EP3 staining was not associated with tumor size (≤ 2 cm vs. > 2 cm, *p* = 0.394), primary lymph node metastasis (pN0 vs. pN1–3, *p* = 0.132) the histological subtype of the tumor (NST vs. non NST, *p* = 0.970), tumor grade (G1-G2 vs. G3, *p* = 0.944) or with the number of tumor foci (unifocal vs. more than one focus, *p* = 0.977). EP3 staining was also not associated with positivity for the hormone receptors ER (*p* = 0.188) or PR (*p* = 0.227) or with HER2-amplification (*p* = 0.716). The distribution of EP3 staining patterns in relation to the different clinicopathological parameters (histology, focality, hormone receptors, HER2-amplification, tumor size, lymph node status, grading, age) is displayed in Table 2.

**Table 2:** Distribution of EP3 staining patterns

		EP3 negative		EP3 positive		
		n	(%)	n	(%)	*p*-value
histology						
	NST	42	(14.5)	102	(35.3)	
	non NST	42	(14.5)	103	(35.6)	0.970
tumor foci						
	unifocal	44	(15.2)	107	(37.0)	
	≥ 2 foci	40	(13.8)	98	(33.9)	0.977
tumor grade						
	G1-G2	31	(20.5)	76	(50.3)	
	G3	13	(8.6)	31	(20.5)	0.944
pT						
	pT1	53	(18.3)	140	(48.4)	
	pT2-pT4	31	(10.7)	65	(22.5)	0.394
pN						
	pN0	42	(14.6)	123	(42.9)	
	pN1-pN3	41	(14.3)	81	(28.2)	0.132
ER						
	negative	12	(4.2)	43	(14.9)	
	positive	72	(24.9)	162	(56.1)	0.188
PR						
	negative	30	(10.4)	89	(30.8)	
	positive	54	(18.7)	116	(40.1)	0.227
HER2						
	negative	70	(24.9)	176	(62.6)	
	positive	11	(3.9)	24	(8.5)	0.716
age (years)						
	median	59.0		57.7		
	range	62		60		0.286

*NST* no special type, *ER* estrogen receptor, *PR* progesterone receptor

### EP3 receptor positivity is significantly associated with improved progression-free survival

**Fig. 2:**
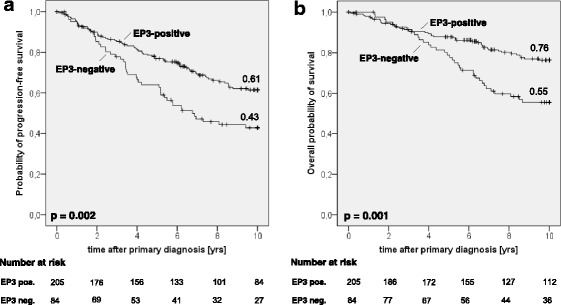
Overall survival and progression-free survival in sporadic breast cancer. Kaplan-Meier estimates of overall probability of survival (**b**) and probability of progression-free survival (**a**) of EP3 positive and negative patient groups are displayed. Estimated survival rates are displayed at the end of each graph, *p*-values in the lower left corner. EP3 positivity was significantly associated with improved probability of progression-free survival (**a**) and improved overall probability of survival (**b**). yrs.: years, neg: negative, pos: positive

Overall, 111 of 289 patients showed progression-free survival after 10 years. 115 of 289 patients suffered from progression during the observation period; for 63 patients, follow-up ended earlier than 10 years. In EP3 negative patients, progression occurred in 45 of 84 patients, while 70 of 205 patients had progressive disease in EP3 positive cases. Kaplan-Meier analysis estimated a 10 years progression-free survival rate of 61% in EP3 positive but only 43% in EP3 negative cases (Fig. 2a, *p* = 0.002); so EP3 positivity was significantly associated with improved progression-free survival. Progression-free survival functions including progression-free survival rates and *p*-values are displayed in Fig. 2a.

Regarding the distribution of distant metastasis and local recurrence as distinct manifestations of progression, the analyzed collective was similar to known collectives of patients with sporadic breast cancer. Kaplan-Meier analysis estimated a 10-year rate of distant metastasis of 26% in EP3 positive but 41% in EP3 negative patients (*p* = 0.012). Similarly, EP3 receptor expression was adversely associated with local recurrence after 10 years (estimated local recurrence rate of 15% in EP3 positive vs. 28% in EP3 negative cases, *p* = 0.016). The difference in progression-free survival between EP3 positive and EP3 negative patients could thus be confirmed in both main parameters of progression, metastasis as well as local recurrence. Kaplan-Meier-estimates of distant metastasis and local recurrence are shown in (see Additional file 1: Figure S1).

### EP3-receptor positivity is significantly associated with improved overall survival

Overall, 147 of 289 patients were alive after 10 years. 78 of 289 patients died during the observation period; follow up ended earlier than 10 years for 64 patients. In EP3 negative cases, 35 deaths occurred (of 84 patients); in EP3 positive patients, 43 of 205 patients died. The estimated overall-survival rate after 10 years by Kaplan-Meier analysis was 76% in EP3 positive but only 55% in EP3 negative cases (*p* = 0.001, Fig. 2b); so EP3 positivity was significantly associated with improved overall survival.

Overall survival functions including overall survival rates and *p*-values are displayed in Fig. 2b.

### EP3-receptor expression is a significant prognosticator for overall survival and progression-free survival

To determine the prognostic relevance of EP3-receptor expression when other prognosticators were taken into account, we performed multivariate Cox regression analysis as described above (methods, statistics).

EP3-receptor positivity was observed to be a prognostic factor for improved progression-free survival (*p* = 0.003, hazard ratio [HR] = 1.81, 95% confidence interval [CI] 1.22–2.67) and improved overall survival (*p* = 0.002, HR = 2.16, 95% CI 1.32–3.53) even when the effects of further prognostic variables (named in Table 3) were accounted for. Besides EP3 positivity, a tumor size smaller than 2 cm and ER positivity were further prognostic factors associated with advanced progression-free and overall survival in the multivariate analysis. Additionally, patient age (continuous) and PR positivity were prognostic factors for overall survival. Data of the multivariate analysis is shown in Table 3. Schoenfeld residuals of EP3 are shown in Additional file 2: Figure S2.

**Table 3:** Multivariate Cox regression analysis of progression-free and overall survival

Covariate	Progression-free survival					Overall survival				
			95 % CI					95 % CI		
	Coefficient	HR	Lower	Upper	*p*-value	Coefficient	HR	Lower		*p*-value
	(B)	Exp(B)				(B)	Exp(B)			
age (continous, unit: 1 year)	0.008	1.009	0.99	1.02	0.254	0.04	1.04	1.02	1.06	*<0.001
pT2-4, reference category: pT1	-0.879	0.42	0.28	0.62	*<0.001	-1.17	0.31	0.19	0.52	*<0.001
pN1-3, reference category: pN0	-0.21	0.81	0.53	1.23	0.323	-0.44	0.64	0.38	1.10	0.107
ER pos, reference category: ER neg	0.52	1.69	1.08	2.63	*0.021	0.75	2.11	1.19	3.74	*0.011
PR pos, reference category: ER neg	n.i.					0.56	1.75	1.03	2.98	*0.040
HER2 pos, reference category: HER2 neg	n.i.					-0.57	0.57	0.31	1.04	0.067
EP3 pos, reference category: EP3 neg	0.59	1.81	1.22	2.67	*0.003	0.77	2.16	1.32	3.53	*0.002
number of samples	275					275				
number of events	108					72				

*HR* hazard ratio, *CI* confidence interval, *Pos* positive, *Neg* negative, *n.i.* not included in multivariate model, as *p*>0.10 in bivariate analysis, *statistically significant (*p*-value < 0.05)

## Discussion

Data from literature show COX-2 overexpression in breast cancer resulting in elevated PGE_2_ synthesis which is thought to contribute to disease progression. Recent studies have evaluated the mechanisms through which PGE_2_ exerts its effects in tumorigenesis and have shown that the expression of PGE_2_ receptors EP1–4 is modified in different kinds of cancer. EP2 and EP4 expression is rather associated with an unfavorable outcome, whereas data regarding the role of the other EP receptors EP1 and EP3, especially in breast cancer, is still sparse [4]. However, as EP3 has the unique feature that it mainly signals via an inhibitory pathway (EP2 and EP4 on the contrary activate a stimulatory pathway), its role in breast cancer and its eligibility as a possible therapeutic target should not be neglected.

This study was performed to analyze the prognostic relevance of EP3 receptor expression in sporadic breast cancer and its association with clinicopathological tumor characteristics (e. g. tumor size, lymph node status, hormone receptors, histology).

We have confirmed that in the majority of sporadic breast cancer cases, EP3 receptor was expressed like it was shown e. g. for different inflammatory breast cancer cell lines [21]. EP3 expression occurred in all histological subtypes of breast cancer and the expression did not differ between the histological subtypes. Therefore, targeting EP3 diagnostically or therapeutically seems generally possible and could be applied to any histological breast cancer subtype. However, EP3 expression was not compared between healthy tissue and tumor – published studies have shown a downregulation of EP3 in breast cancer compared to healthy breast tissue [8], in colon cancer [22] and in prostate cancer [23].

EP3 receptor expression was independent of other clinicopathological factors (named in Table 1) which partly have known prognostic relevance (like e.g. ER-positivity). COX-2 overexpression in breast cancer on the contrary is mostly associated with clinicopathological factors characteristic for an aggressive phenotype, like large tumor size, negative hormone receptor status or high proliferation [6, 24]. Other studies, however, did not show an association of COX-2 overexpression with clinicopathological parameters [25, 26].

Interestingly, EP3 receptor expression was not associated with an unfavorable course of the disease, like it is known e. g. from EP2 and EP4 [13, 14, 17, 27]. On the contrary, instead of a negative influence of EP3, an association of EP3 with improved survival and improved progression-free survival could be shown. EP3 was even a significant prognostic factor when other factors with known prognostic influence were accounted for (Table 3). To our knowledge, this is the first report of EP3 as a prognosticator for survival or progression-free survival in breast cancer.

Data concerning EP3 in breast cancer is sparse. EP3 is described as irrelevant in one study, where treatment of breast cancer cells with EP3 antagonists had no effect on metastasis [15]. Another study in inflammatory breast cancer, however, showed a beneficial effect of EP3, as treatment of inflammatory breast cancer cells with the EP3 agonist sulprostone reduced the ability of the tumor cells to undergo vasculogenic mimicry, a characteristic of very aggressive tumor types [21].

Regarding the role of EP3 in other cancer types, both pro- and anti-tumorigenic effects have been described.

Some data suggest a pro-tumorigenic effect of EP3 receptor expression, as EP3 has been associated to angiogenesis and lymphangiogenesis: When Lewis lung carcinoma cells were injected in mice, tumor-associated angiogenesis, metastasis and tumor growth were reduced in EP3 knockout mice compared to wild type mice. Also, levels of vascular endothelial growth factor, matrix-metalloproteinase-9 and of podoplanin, a marker for lymphatic endothelial cells, were reduced in EP3 knockout mice and induced by EP3 agonists [28–30].

Other studies, on the contrary, suggest an anti-tumorigenic effect of EP3 receptor expression. EP3 knockout e.g. enhanced azoxymethane-induced colon carcinogenesis [22] and contributed to squamous cell carcinoma development [31]. Similarly, EP3 knockdown in prostate cancer cells or treatment of prostate cancer cells with EP3 antagonists accelerated tumor cell growth [32]. Consistent with this data, EP3 overexpression in prostate cancer cells impaired tumor growth in vitro and stimulation of cells overexpressing EP3 with the EP3 agonist sulprostone further enhanced the inhibitory effect [23]. Therefore, the authors of these studies hypothesize that the reduction of EP3 expression during tumorigenesis might be consistent with tumor-suppressive properties of EP3.

Differences between the above named studies might be partly due to the different EP3 isoforms, as EP3 isoforms mainly signal via Gi proteins but partly also via the Ca^2+^/phospholipase cascade or via Gs proteins [4]. Therefore, different effects in studies concerning EP3 might be to some extent due to different expression patterns of the isoforms. In prostate cancer e.g., EP3 II, an EP3 isoform coupled to Gi protein, was the major isoform found and EP3-expression also showed inhibitory effects on tumor cell growth in this study [23]. In mice, three EP3 isoforms exist and overexpression of all three variants has been associated with reduced tumor cell growth [33]. For future studies in breast cancer it is therefore necessary to clarify which isoforms are expressed in which manner to better understand the effects and to facilitate comparing different studies.

In summary, our study has shown that in breast cancer, EP3 was a significant prognosticator for improved progression-free and overall survival without association of its expression to known clinicopathological parameters. This is contrary to part of the above named studies, where EP3 has shown negative effects and is also contrary to the negative effects of the other EP receptors (EP2, EP4) in breast cancer [4]. The positive prognostic influence of EP3 in breast cancer is surprising insofar, as both COX-2 overexpression and PGE_2_ elevation have been shown to have pro-tumorigenic effects in breast cancer, and EP3 is part of the signaling pathway of PGE_2_ and is therefore mediating PGE_2_ effects. As EP3 seems to have a beneficial effect in breast cancer, it is likely, that one or more of the functional effects of PGE_2_ in tumorigenesis (proliferation, invasiveness, metastasis, anti-apoptotic effect) might be inhibited by EP3. Our future work will concentrate on identification of the factor that is regulated by EP3. By improving the understanding of the functional aspects of EP3 and its regulated factors, we aim to evaluate its eligibility as a possible future target in breast cancer prevention and treatment.

## Conclusions

In conclusion, this study shows that EP3-receptor is a significant prognosticator for improved progression-free and overall survival in sporadic breast cancer. This opens the possibility to use EP3 as a prognostic factor but also to further examine it as a target for future specific anti-tumor therapies. Therefore, the mechanisms how EP3 suppresses tumor progress have to be evaluated in future research.

## Additional files

Additional file 1: **Figure S1.** Metastasis and local recurrence in sporadic breast cancer. 10-years Kaplan-Meier-estimates of cumulative metastasis (A) and cumulative local recurrence (B) of EP3 positive and negative patient groups are displayed. Estimated metastasis and local recurrence rates are displayed at the end of each graph, *p*-values in the upper left corner. EP3 positivity was significantly associated with reduced metastasis (A) and local recurrence (B). Note that in the rates of metastasis and local recurrence named there, all cases of metastasis/local recurrence are considered, regardless if they were the primary cause of progression or happened later in the course of disease; therefore, the sum of both rates here is higher than the progression rate named in Fig. 2. yrs. = years. (PDF 437 kb)

Additional file 2: **Figure S2.** Schoenfeld residuals for EP3. To test for PH assumption, Schoenfeld residual test was performed for EP3 for OS and PFS. Schoenfeld residuals for EP3 are displayed (left: OS, right: PFS). There is no violation of PH assumption, as the ratio of both curves is stable over the time. (PDF 20 kb)

## Abbreviations

cAMP: Cyclic adenosine monophosphate
CI: Confidence interval
COX: Cyclooxygenase
COXib: COX-2 antagonist
ELISA: Enzyme-linked immunosorbent assay
ER: Estrogen receptor
HER2: Human epidermal growth factor receptor 2
HR: Hazard ratio
IRS: Immunoreactivity score
NST: No special type
PG: Prostaglandin
PR: Progesterone receptor
yrs.: Years
