# Supplementary material for: EP3 (prostaglandin E2 receptor 3) expression is a prognostic factor for progression-free and overall survival in sporadic breast cancer
Source: BMC Cancer. 2018 Apr 16;18:431. doi: 10.1186/s12885-018-4286-9 (PMC5902996; doi:10.1186/s12885-018-4286-9)
Supplement: Supplementary file 1 — Figure S1. Metastasis and local recurrence in sporadic breast cancer. 10-years Kaplan-Meier-estimates of cumulative metastasis (A) and cumulative local recurrence (B) of EP3 positive and negative patient groups are displayed. Estimated metastasis and local recurrence rates are displayed at the end of each graph, p-values in the upper left corner. EP3 positivity was significantly associated with reduced metastasis (A) and local recurrence (B). Note that in the rates of metastasis and local recurrence named there, all cases of metastasis/local recurrence are considered, regardless if they were the primary cause of progression or happened later in the course of disease; therefore, the sum of both rates here is higher than the progression rate named in Fig. 2. yrs. = years. (PDF 437 kb) [file 12885_2018_4286_MOESM1_ESM.pdf]

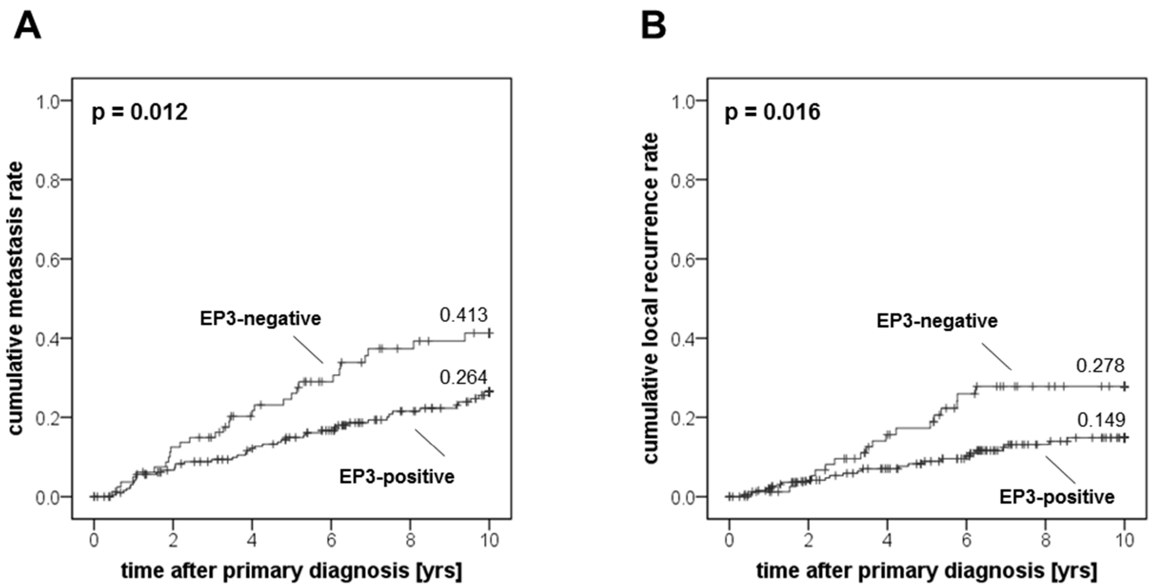


**Figure S1:** Metastasis and local recurrence in sporadic breast cancer. 10-years Kaplan-Meier-estimates of cumulative metastasis (**A**) and cumulative local recurrence (**B**) of EP3 positive and negative patient groups are displayed. Estimated metastasis and local recurrence rates are displayed at the end of each graph, p-values in the upper left corner. EP3 positivity was significantly associated with reduced metastasis (**A**) and local recurrence (**B**). Note that in the rates of metastasis and local recurrence named there, all cases of metastasis/local recurrence are considered, regardless if they were the primary cause of progression or happened later in the course of disease; therefore, the sum of both rates here is higher than the progression rate named in figure 2. yrs = years.
