# Supplementary figures and images for: EP3 (prostaglandin E2 receptor 3) expression is a prognostic factor for progression-free and overall survival in sporadic breast cancer
Source: BMC Cancer. 2018 Apr 16;18:431. doi: 10.1186/s12885-018-4286-9 (PMC5902996; doi:10.1186/s12885-018-4286-9)

**Schoenfeld residuals of EP3**

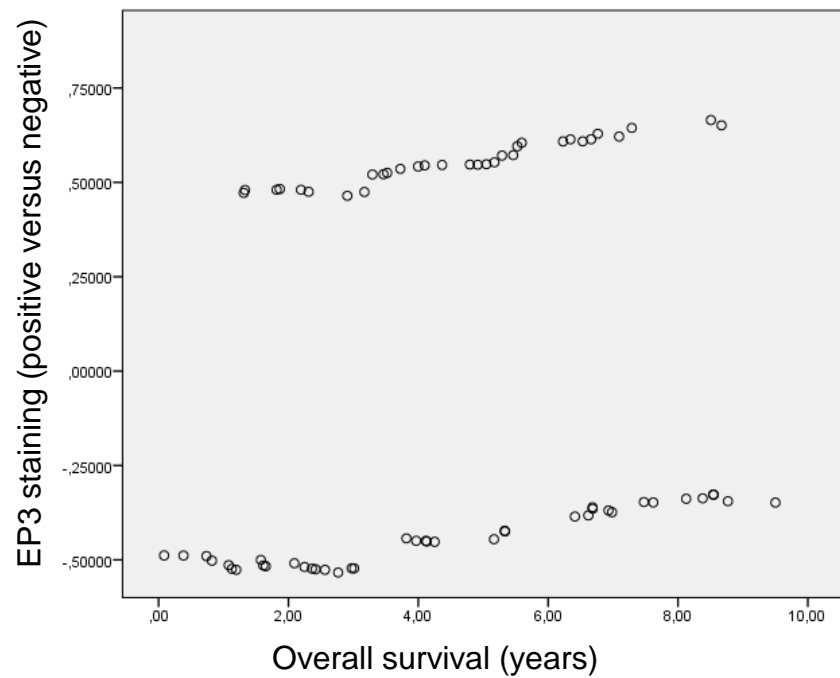

**Schoenfeld residuals of EP3**

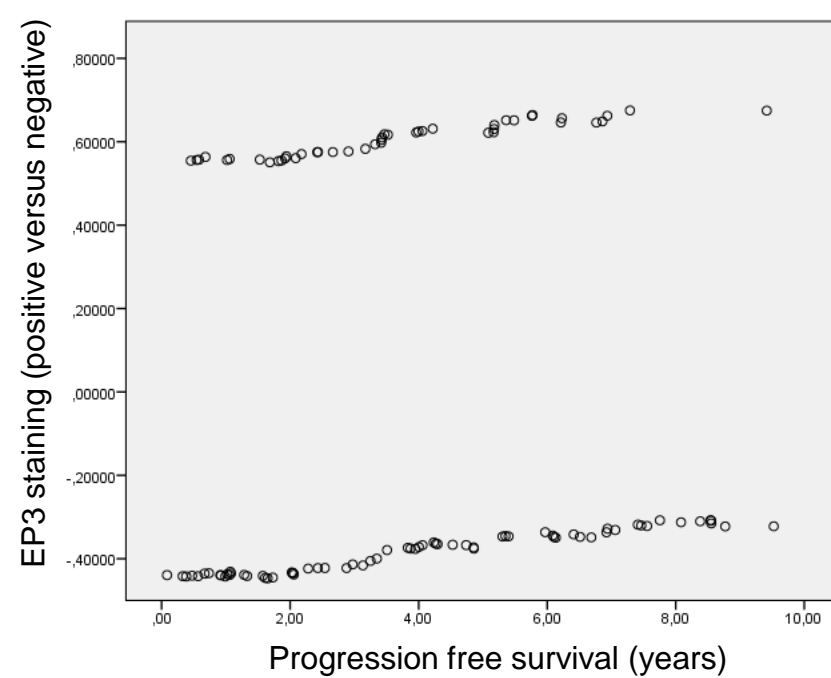

Supplement: Supplementary file 2 — Figure S2. Schoenfeld residuals for EP3. To test for PH assumption, Schoenfeld residual test was performed for EP3 for OS and PFS. Schoenfeld residuals for EP3 are displayed (left: OS, right: PFS). There is no violation of PH assumption, as the ratio of both curves is stable over the time. (PDF 20 kb) [file 12885_2018_4286_MOESM2_ESM.pdf]
